# Major vault protein attenuates cardiomyocyte injury in doxorubicin-induced cardiomyopathy through activating AKT

**DOI:** 10.1186/s12872-022-02517-9

**Published:** 2022-03-04

**Authors:** Yu Qi, Jianzhou Chen, Junfeng Duan, Lina Kang, Kun Wang, Ziwei Chen, Biao Xu, Rong Gu

**Affiliations:** 1 Department of Cardiology, Nanjing Drum Tower Hospital, Affiliated Hospital of Nanjing University Medical School, Nanjing, 210008 China; 2 Department of Cardiology, Affiliated Hospital of Nantong University, Nantong, 226001 China; 3 State Key Laboratory of Pharmaceutical Biotechnology, Nanjing University, Nanjing, 210023 China

**Keywords:** Doxorubicin-induced cardiomyopathy, Major vault protein, AKT, Apoptosis

## Abstract

**Background:**

Doxorubicin (DOX) has limited chemotherapy application for malignancies due to cardiotoxicity. The pathogenesis of DOX-induced cardiomyopathy (DiCM) is yet to be elucidated. Increasing studies proved that activation of AKT prevented cardiomyocyte apoptosis and cardiac dysfunction in response to DOX insult. Our previous studies indicated that major vault protein (MVP) deficiency was accompanied by suppressed phosphorylation of AKT in metabolic diseases. This study aimed to investigate the role and underlying mechanism of MVP on cardiomyocyte apoptosis in DiCM.

**Methods:**

Mice were intraperitoneally injected with DOX 5 mg/kg, once a week for 5 weeks, the total cumulative dose was 25 mg/kg. Cardiomyocyte-specific MVP overexpression was achieved using an adeno-associated virus system under the cTnT promoter after the fourth DOX injection. Cardiac function was examined by echocardiography followed by euthanasia. Tissue and serum were collected for morphology analysis and biochemical examination.

**Results:**

Herein, we found that MVP expression was upregulated in DOX-treated murine hearts. Cardiac-specific MVP overexpression alleviated DOX-induced cardiac dysfunction, oxidative stress and fibrosis. Mechanistically, MVP overexpression activated AKT signaling and decreased cardiomyocyte apoptosis in DiCM.

**Conclusions:**

Based on these findings, we supposed that MVP was a potential therapeutic agent against DiCM.

**Supplementary Information:**

The online version contains supplementary material available at 10.1186/s12872-022-02517-9.

## Background

Doxorubicin (DOX) is one of the most highly prescribed anthracyclines due to its broad spectrum of chemotherapeutic efficacy [1]. However, its use can result in a dose-dependent toxicity to cardiomyocytes and develop irreversible degenerative cardiomyopathy and congestive heart failure, which greatly limits DOX clinical application for malignancies [2, 3]. A series of studies have been demonstrated that transcriptional dysregulation, Ca^2^^+^ abnormalities and mitochondrial dysfunction are chief among the molecular and cellular mechanisms of a cumulative dose of DOX-induced cardiomyopathy (DiCM) [4]. Indeed, whatever the driver of cardiomyocyte bioenergetic failure, the progressive cardiomyocyte loss with the activation of apoptosis signaling pathway is the key consequence of DOX insult [5]. Thus, reducing apoptosis of cardiomyocytes is a promising therapeutic strategy for DiCM and a way to prevent heart failure.

AKT, or protein kinase B (PKB), is a 57-kDa serine/threonine-protein kinase which belongs to the cAMP-dependent protein kinase A/G/C superfamily [6]. Normally, inactive form of AKT resides in the cytoplasm, while activated AKT, phosphorylated form (pAKT), will translocate from the phosphorylated site in plasma membrane to nucleus to activate a wide range of substrates involving multiple cellular processes such as cell survival, proliferation, apoptosis and metabolism [7]. AKT is a well-characterized candidate of phosphoinositide 3-kinase (PI3K) in PI3K/AKT/mTOR signaling pathway medicating cell survival responses in cancer [8], as well as a key point in PTEN/AKT/p53/cyclinD1 underlying tumorigenesis [9]. Increasing studies have revealed that activation of AKT can prevent cardiomyocyte apoptosis and cardiac dysfunction [10, 11], while little is known about of the positive regulator of it in DiCM.

Major vault protein (MVP), also called lung resistance-related protein (LRP), is the dominant structural protein of the vault complex, which is a hollow, barrel-like ribonucleoprotein particle in the cytoplasm and is widely expressed [12]. MVP plays a role in multidrug resistance, autophagy, and cell signaling and is widely researched in the tumor area [13]. The elements of MVP promoter contain binding sites for transcription factors, including the tumor suppressor protein p53, which inversely associated with the occurrence of carcinoma [14]. In addition,. Besides, our previous studies showed that MVP deficiency was accompanied by suppressed phosphorylation of AKT in metabolic diseases [15]. However, it is unknown whether MVP is required for the development of DiCM or how MVP might protect the cardiac function. In this study, we identify that MVP was upregulated in the heart tissue of DiCM mouse model. Adeno-associated virus serotype 9 (AAV9)-mediated overexpression of MVP significantly attenuates cardiomyocyte apoptosis in DiCM through activating AKT, providing a potential cardioprotective target to improve cardiac function from DOX-induced cardiomyopathy.

## Methods

### Mice model

Forty C57BL/6 male mice (7–8 weeks old, 22–26 g) were purchased from the Model Animal Research Center of Nanjing University (GemPharmatech, Nanjing, China) and the experiment lasted about one year. Mice were housed at 22–24 °C under standard light conditions (12 h light/dark cycle) and subjected to an adaptive feeding for 1 week before the study commenced.

**Fig. 1:**
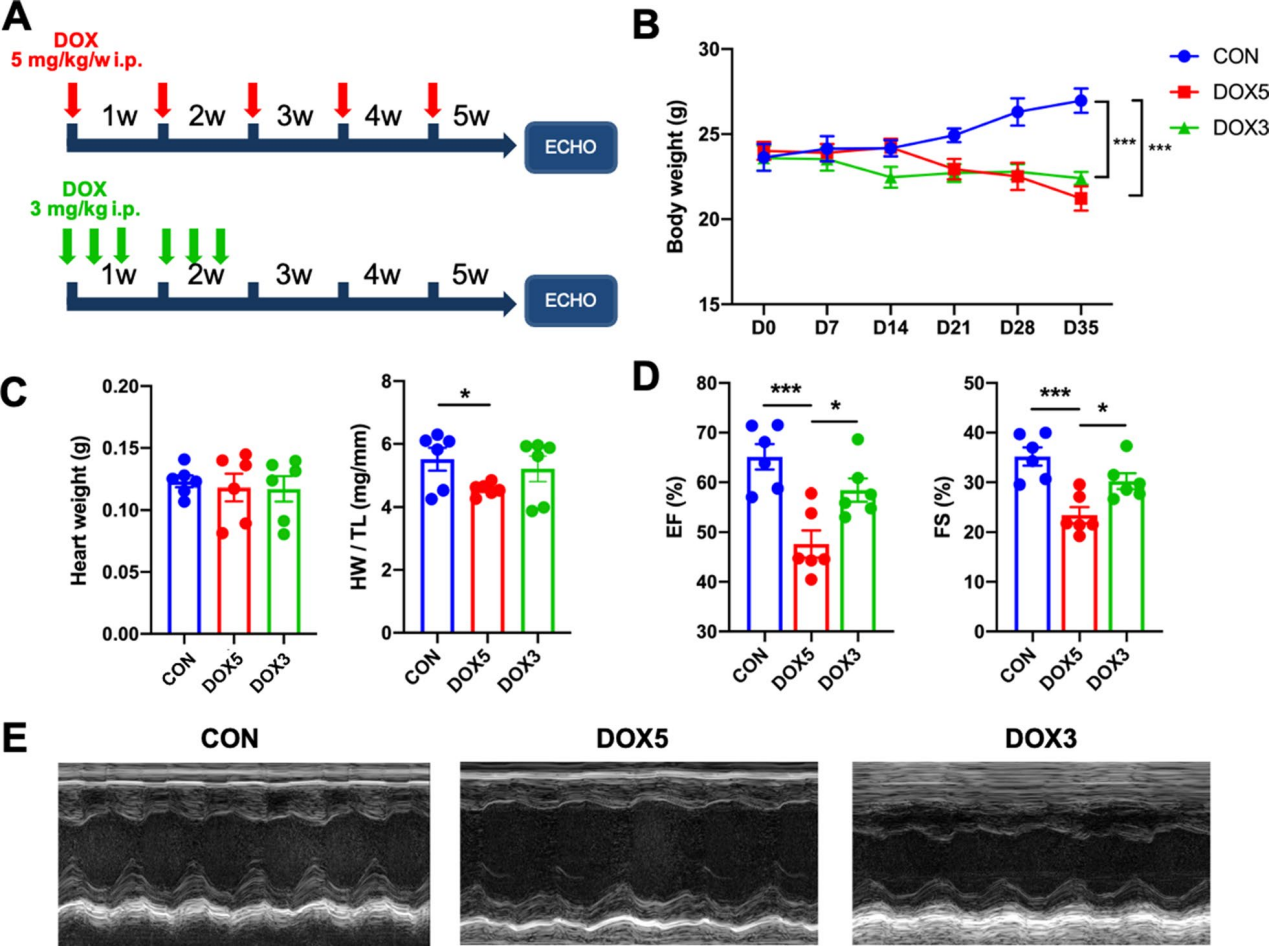
Effects of doxorubicin-induced cardiotoxicity in mice. **A** Flow chart of the chronic DiCM models. ECHO, echocardiography. **B** Bodyweight alterations. **C** Statistical results of the heart weight (HW) and HW/tibia length (TL). **D**, **E** Percentage of the ejection fraction (EF) and fractional shortening (FS) of mice as determined via echocardiography. N = 6, values represent the mean ± SEM. *P < 0.05, **P < 0.01, ***P < 0.001

For identify a suitable DOX concentration of chronic DOX (S1208, Selleck)-induced heart failure model, mice were divided into three groups (n = 6): (1) In CON group, mice were treated with saline containing 0.5% dimethyl sulfoxide (DMSO) triple a week for two weeks; (2) In DOX5 group, DOX (5 mg/kg) was administered intraperitoneally (i.p.) with once a week for a total cumulative dose of 25 mg/kg; (3) In DOX3 group, DOX (3 mg/kg) were administered i.p. thrice a week for a total cumulative dose of 18 mg/kg. Bodyweight was measured weekly and cardiac function was assessed by echocardiography (ECHO) (Fig. 1A). In general, mice treated with DOX 5 mg/kg showed significant decrease in cardiac function and this concentration was used in the following study.

To investigate that whether specifical overexpress MVP in myocardium has therapeutic significance for DiCM, mice were divided into three groups (n = 7, 8, 7, respectively): (1) In Con group, mice were treated with saline containing 0.5% DMSO once a week for 5 weeks; (2) In DOX + Vec group (negative control), mice first received DOX 5 mg/kg once a week and then treated with a single intravenous (i.v.) injection of  AAV9 vector via the tail vein at a titer of 1 × 10^12^ viral genome (v.g.) per mouse immediately after the fourth DOX injection; (3) In DOX + MVP group, DiCM mice model were same to the above, but received AAV9 carrying mouse MVP under the cTnT promoter (Fig. 3B).

The AAV9-cTnT-MVP and AAV9-cTnT-Vector were generated and purification by Viraltherapy Technologies Co. (Wuhan, China). After 8 weeks from the first injection of DOX, cardiac function was evaluated and murine hearts and serum were collected for further detection.

### Echocardiography

The mice were anesthetized with 1% isoflurane and monitored for respiratory frequency and temperature. Cardiac function assessed by transthoracic echocardiography (Vevo 2100; Visual Sonics) was performed according to our previous studies [16]. In brief, left ventricular (LV) dimensions, including diastolic and systolic wall thickness and LV end-diastolic and end-systolic chamber dimensions, were measured from 2D short-axis under M-mode tracings at the level of the papillary muscle. The left ventricular ejection fraction (EF) and left ventricular fractional shortening (FS) were calculated from 3 to 5 cardiac cycles.

### Histology assay

Mice were euthanized and euthanized, hearts and serum were collected as described [17]. In brief, hearts were harvested and fixed in 4% paraformaldehyde overnight, embedded in O.C.T. (SAKURA, 4583) with the apex toward the surface. Then cut into 5-μm-thick transversal sections serially. The sections were stained with hematoxylin and eosin (HE) to analyze the global heart morphology and inflammatory cell infiltration and stained with Masson’s trichrome to measure collagen deposits. The sections were scanned at 20-fold magnification on a high-resolution microscope (Leica, Japan), and the images of Masson staining of perivascular were captured at 40-fold magnification.

### Immunohistochemistry (IHC) and terminal deoxynucleotidyl transferase- mediated dUTP nick end labeling (TUNEL) staining

IHC staining was performed according to our previous studies[18]. Nonspecific binding of the antibody was blocked with 5% donkey serum (S30, Millipore) in 1 h. The MVP antibody (1:200 dilution, sc-18701, Santa Cruz) was used to determine the protein expression levels. Cryosections of heart were also processed for TUNEL staining according to the manufacturer’s instruction (12156792910, Roche) and covered the slides with DAPI Fluoromount-G (0100-20, SouthernBiothch). The histological features were observed and captured in 20 or 40-fold magnification.

### Transmission electron microscopy (TEM)

For TEM images, mice heart apex pieces were fixed in 1% glutaraldehyde in PBS, overnight at 4 °C. Samples were post-fixed in 1% osmium tetroxide and dehydrated through a series of ethanol solutions. After the last dehydration step, samples were incubated in a 1:3, 1:1, 3:1 mixture of Durcupan resin and acetone and cured at 60 °C for 48 h. Ultrathin sections (50–60 nm) were obtained using a diamond knife (Diatome) in an ultramicrotome (Leica Reichert ultra-cut S) and then collected. The sections were counterstained with 2% uranyl acetate in water for 20 min followed by a lead citrate solution. Samples were examined with a JEOL JEM1010 electron microscope (Tokyo, Japan) equipped with an Orius SC200 digital camera (Gatan Inc.) at the Transmission Electron Microscopy Laboratory in Nanjing Medical University.

### Oxidative stress measurements and biochemical determination

Peripheral blood samples were collected 8 weeks after the first DOX injection. Malondialdehyde (MDA) and superoxide dismutase (SOD) levels in serum were measured using kits (Jiancheng Bio, China) according to the manufacturer’s instructions. The concentrations of lactate dehydrogenase (LDH) and creatine kinase isoenzymes (CK-MB) were measured by an automatic biochemical analyzer (ADVIA 2400, China).

### Cell culture

H9C2 cells were purchased from the Cell Bank of the Chinese Academy of Sciences (Shanghai, China) and were cultured in DMEM medium with 10% fetal bovine serum. After synchronization for 12 h, cells were treated with DOX (2 mM) for 24 h.

### Quantitative RT-PCR

Total RNA was extraction from heart tissues using RNAiso Plus (9109, Takara, Japan) according to the manufacturer’s protocol. The concentration of RNA obtained was measured using a supersensitive NanoDrop OD-2000 spectrophotometer. Reverse transcription reaction was performed using the SuperMix (R323-01, Vazyme Biotech, China). Quantitative RT-PCR was performed using the SYBR Green (Q711-01, Vazyme Biotech, China) on a Quant Studio 6 system (Thermo Fisher Scientific, USA) with standard PCR conditions. The expression level of each transcript was normalized to glyceraldehyde-3-phosphate dehydrogenase (GAPDH). All samples were run in triplicate and the relative expression level for each mRNA was calculated using the 2^−ΔΔCt^ method. Primers are listed in Table 1.

**Table 1:** The sequences of PT-PCR primers

Primer		Sequence (5′ to 3′)
MVP	Forward	TGGATCTGGTGGACGCTGTGAT
	Reverse	TGCACCGTCACTAGCCATTCCT
Trp53inp1	Forward	TGCACCGTCACTAGCCATTCCT
	Reverse	TAAGCAGCTTTTTCTGGCCCT
Ppargc1a	Forward	GCACGCAGCCCTATTCATTG
	Reverse	TGAGTCTCGACACGGAGAGT
Ppargc1b	Forward	AGAAGGTTGGCTGACATGGG
	Reverse	AGGTCAAGCTCTGGCAAGTC
GAPDH	Forward	GGGTCCCAGCTTAGGTTCAT
	Reverse	CCAATACGGCCAAATCCGTT

### Western blot

Heart tissue was quickly homogenized immediately in RIPA buffer (P0013B, Beyotime) with EDTA-free (HY-K0010, MCE) and PhosStop (04906837001, Roche) at 4 °C. After protein-concentration measurement, the lysate was stored at − 80 °C for western blot analysis. Lysate protein (20 μg) was electrophoresed and transferred to polyvinylidene difluoride membranes. After blocking with 5% nonfat dry milk or BSA, the membrane was incubated with primary antibodies at 4 °C overnight. The following antibodies were used: MVP (Santa Cruz, sc-23916, 1:800), phospho-AKT (Cell Signaling, 9271, 1:1000), AKT (Cell Signaling, 9272, 1:1000), Bcl-2 (Abcam, ab182858, 1:1000), Bax (Abcam, ab32503, 1:1000), cleaved Caspase 3 (Cell Signaling Technology, 9661 s, 1:1000), Caspase 3 (Abcam, ab18487, 1:1000), GAPDH (Proteinthch, 10494-1-AP, 1:5000). Full-unedited gel blots could been seen in Additional file 1 in Supplementary Information. 

### Statistical analysis

Data are presented as the mean ± standard error of the mean (SEM). Differences between groups were analyzed with the student’s t-test for two groups or one-way analysis of variance (ANOVA) followed by Tukey post hoc test for multiple groups. Survival data were assessed by the Kaplan–Meier method. Tests were performed using GraphPad Prism 8.0, P < 0.05 was considered statistically significant.

## Results

### Effects of doxorubicin-induced cardiotoxicity in mice

To determine the toxic effect of doxorubicin (DOX) on cardiomyocytes, mice were treated with DOX 5 mg/kg via i.p. once a week for 5 times (DOX5 group), or 3 mg/kg three times a week for two weeks (DOX3 group) as shown in Fig. 1A. The total cumulative dose is 25 or 18 mg/kg respectively [16, 19]. We observed that DOX injection gradually decreased the body weight in two groups, but only the DOX5 group was moderately and effectively enlarge the final ratio of the heart to the tibia length (Fig. 1B, C), which is consistent with the previous studies which indicated that DOX application significantly decreased the body weight in cancer patients [20]. Besides, at 5 weeks after the first DOX injection, we measured cardiac function by echocardiography, then collected the hearts for analysis. DOX5 group did significantly attenuated the heart function compared with the control (CON) group or DOX3 group (Fig. 1D, E), indicated that the DOX5 group made the hypertrophy model successfully. Therefore we selected this dose for the next experiments.

### Expression of MVP is up-regulated in DOX-induced heart tissues

**Fig. 2:**
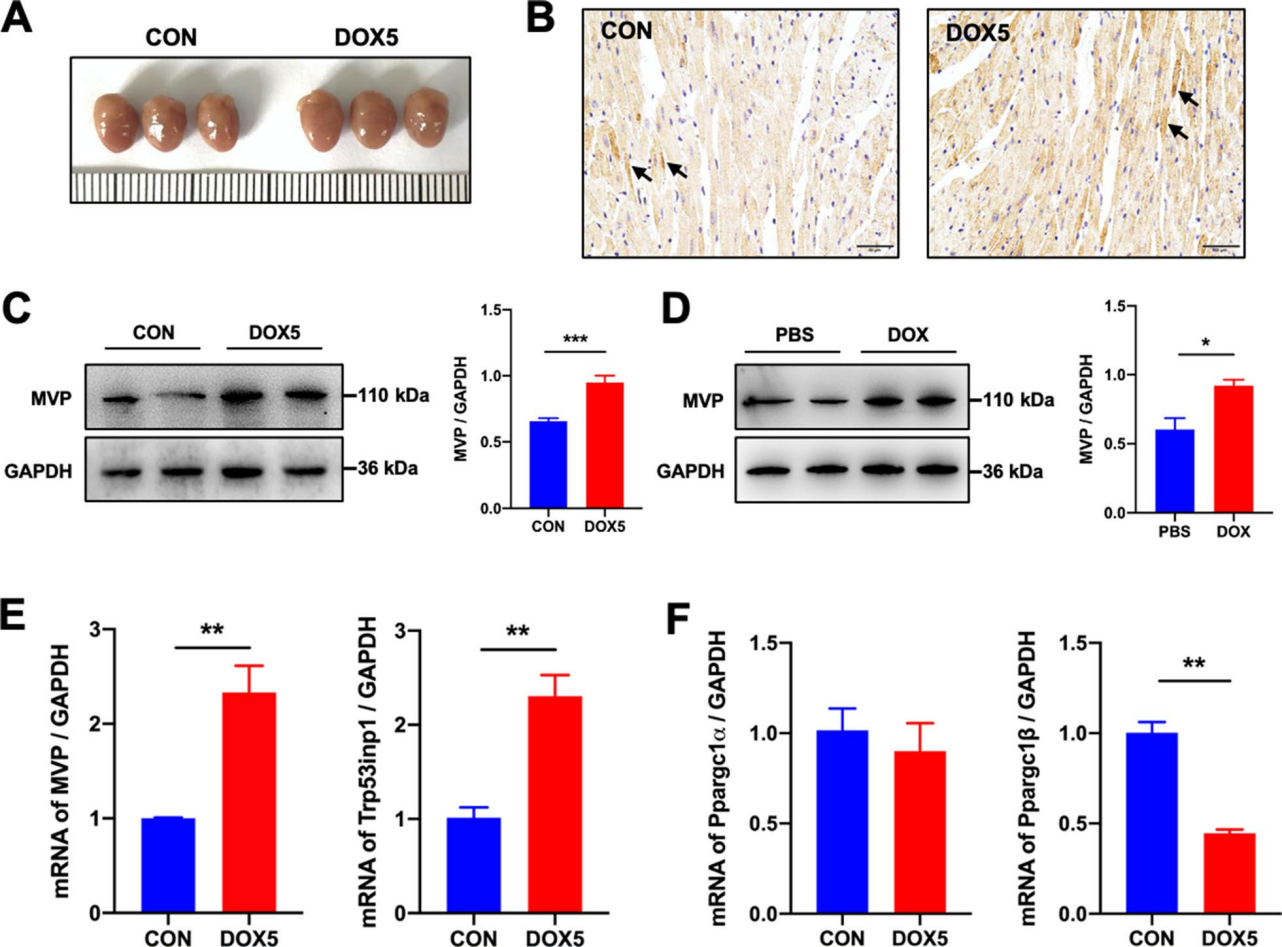
Expression of MVP is up-regulated in DOX-treated heart tissues and cardiomyocytes. **A** The morphology of the hearts of the two groups. **B** Representative immunohistochemistry images of MVP in murine hearts. **C** Western blot and a quantitative result of MVP in DOX-treated hearts. **D** Western blot and a quantitative result of MVP in H9C2 cardiomyocytes (three independent experiment). **E**, **F** The mRNA levels of MVP/p53/Ppargc1a/Ppargc1b in DOX-treated hearts. N = 6, values represent the mean ± SEM. *P < 0.05, **P < 0.01, ***P < 0.001

The morphology of the hearts was slightly enlarged in the DOX5 group compared with the CON group (Fig. 2A). To evaluate the participation of MVP in DiCM, we first determined the expression of MVP. Immunohistochemistry staining showed that DOX caused a significant increase of MVP expression level in the heart tissue compared with the CON group (Fig. 2B), which was further confirmed by the western blot results (Fig. 2C). In addition, DOX incubation upregulating MVP expression was also verified in H9C2 cardiomyocytes (Fig. 2D). The differential expression pattern of MVP was reproduced in the mRNA level with the Trp53inp1 upregulation, a p53-inducible gene that regulates apoptosis (Fig. 2E). Both Ppargc1α and Ppargc1β encodes the transcriptional coactivators PGC-1α and PGC-1β, which has a crucial role in mitochondrial biogenesis. Quantitative PCR analysis confirmed that Ppargc1β transcripts were downregulated strongly in the DOX5 group than in the CON group (Fig. 2F). Thus, MVP is up-regulated in DiCM accompanied by cardiomyocyte apoptosis.

### Overexpression of MVP attenuated DOX-induced cardiac dysfunction and oxidative stress in mice

**Fig. 3:**
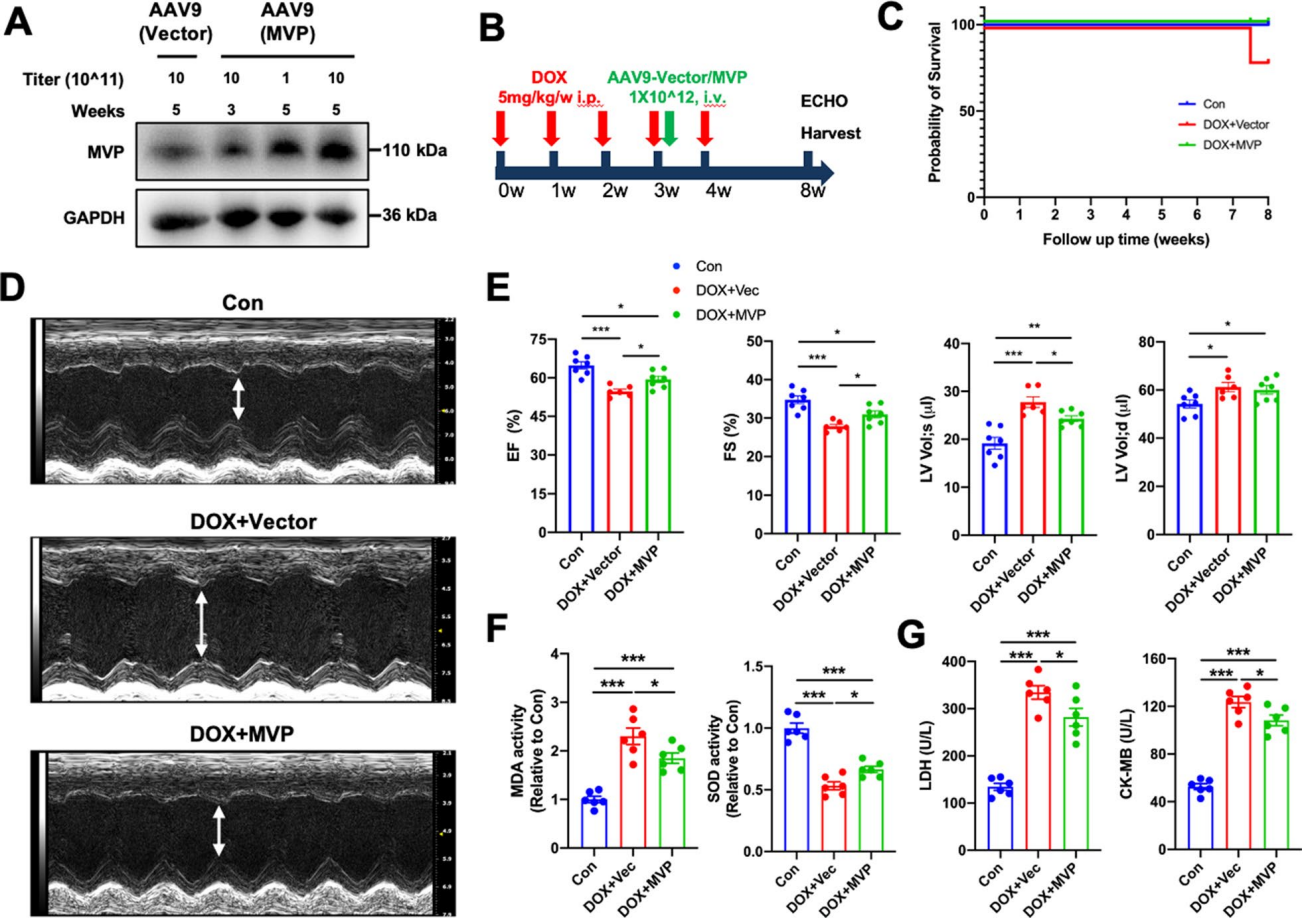
MVP attenuated DOX-induced cardiac dysfunction in mice. **A** Validation of AAV9 overexpression efficiency in vivo. **B** Flow chart of the chronic DiCM models with the administration of AAV9. The heart function was examined 5 weeks after the AAV9 injection followed by euthanasia, and harvest tissues and serum for morphology analysis and biochemical examination. **C** Kaplan–Meier survival curve of mice exposed to repeated injections of both DOX (5 mg/kg, i.p., once a week, the total cumulative dose is 25 mg/kg) and AAV9 (n = 7 or 8). **D**, **E** of mice as determined via echocardiography 8 weeks from the first DOX injection (n = 6 or 7). EF, ejection fraction; FS, fractional shortening; LV Vol;s, left ventricular end-systolic volume; LV Vol;d, LV end-diastolic volume. **F** Activity of the MDA and the antioxidant enzyme SOD in the heart were detected. The data are shown as the fold change relative to the Con group. **G** The levels of LDH and CK-MB in serum 5 weeks after AAV9 injection. N = 6, values represent the mean ± SEM. *P < 0.05, **P < 0.01, ***P < 0.001

To explore the role of MVP in cardiomyocyte for the established DOX-induced heart failure model, we used AAV9 system to specifically overexpressed mouse MVP in the myocardial tissues under the cTnT promoter. First, we assessed the efficiency in vivo, which identified that the efficiency of AAV9 (titer 1 X 10^13) continued to increase within 5 weeks (Fig. 3A). Then we chose this dose and gave a single injection of AAV9-Vector or AAV9-MVP immediately at the fourth DOX injection (Fig. 3B). Kaplan–Meier survival curve showed that two mice in the DOX + Vec group died at 7–8 weeks which indicated that MVP may improve the survival rate (Fig. 3C). As shown in Fig. 3D–E, MVP overexpression prevented DOX-induced cardiac dysfunction and expansion, as indicated by the ejection fraction (EF), the preserved fractional shortening (FS), left ventricular end-systolic volume (LV Vol;s), and LV end-diastolic volume (LV Vol;d). Oxidative damage is the main cause of DiCM, we identified that MVP overexpression inhibited the abnormal MDA level but preserved SOD level (Fig. 3F). In addition, myocardial injury was assessed by the serum levels of LDH and CK-MB, we observed MVP overexpression could protect DOX-induced myocardial injury (Fig. 3G).

### Overexpression of MVP alleviates DOX-induced cardiac fibrosis in mice

**Fig. 4:**
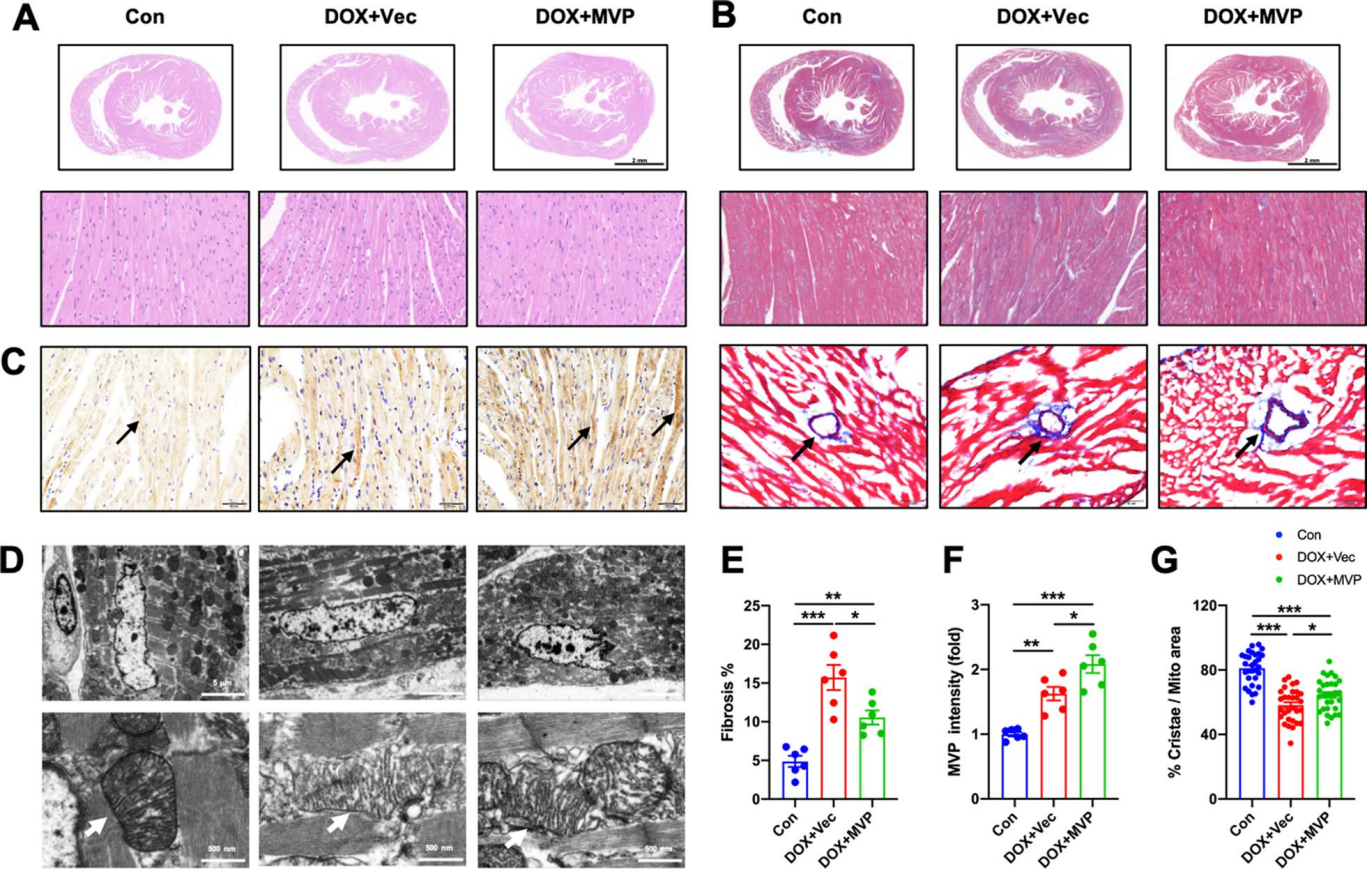
MVP protected DOX-induced cardiac fibrosis mitochondrial injury in mice. **A** Representative images of HE staining of heart are shown 5 weeks after AAV9 injection. **B**, **E** Representative images and the analysis of Masson’s trichrome staining of cardiomyocyte, and the images of perivascular area, are shown 5 weeks after AAV9 injection. **C**, **F** Representative immunohistochemistry images and the analysis of cardiac sections stained with MVP 5 weeks after AAV9 injection. **D** Representative TEM images in mice heart. Mitochondrial can be identified by higher electro density (dark areas). Bottom images show enlarged detail of area in top images that contain mitochondria. **G** Increase mitochondrial fitness in cardiomyocyte in the DOX + MVP group compared with DOX + Vec as defined from TEM images and quantified as percent of cristae area per mitochondrial. Each dot represents one mitochondria. Data are shown as means from 30 mitochondria and 6 mice per group. N = 6, values represent the mean ± SEM. *P < 0.05, **P < 0.01, ***P < 0.001

To assess the heart morphological alterations influenced with MVP overexpression, including ventricular dilation, pathological fibrosis, HE, and Masson staining were performed. Representative images showed that there was significantly less cardiac fibrosis in the DOX + MVP group compared with the DOX + Vector group (Fig. 4A, B, E). The intensity of MVP expression in the DOX + MVP group was higher compared with the DOX + Vec group (Fig. 4C, F). In addition, mitochondria presented conspicuous alterations in morphology featuring increased area and reduced cristae density in response to DOX insult, and MVP overexpression mitigated this pathological alteration (Fig. 4D, G).

### MVP suppressed cardiomyocyte apoptosis in DOX-induced cardiomyopathy through activating AKT

**Fig. 5:**
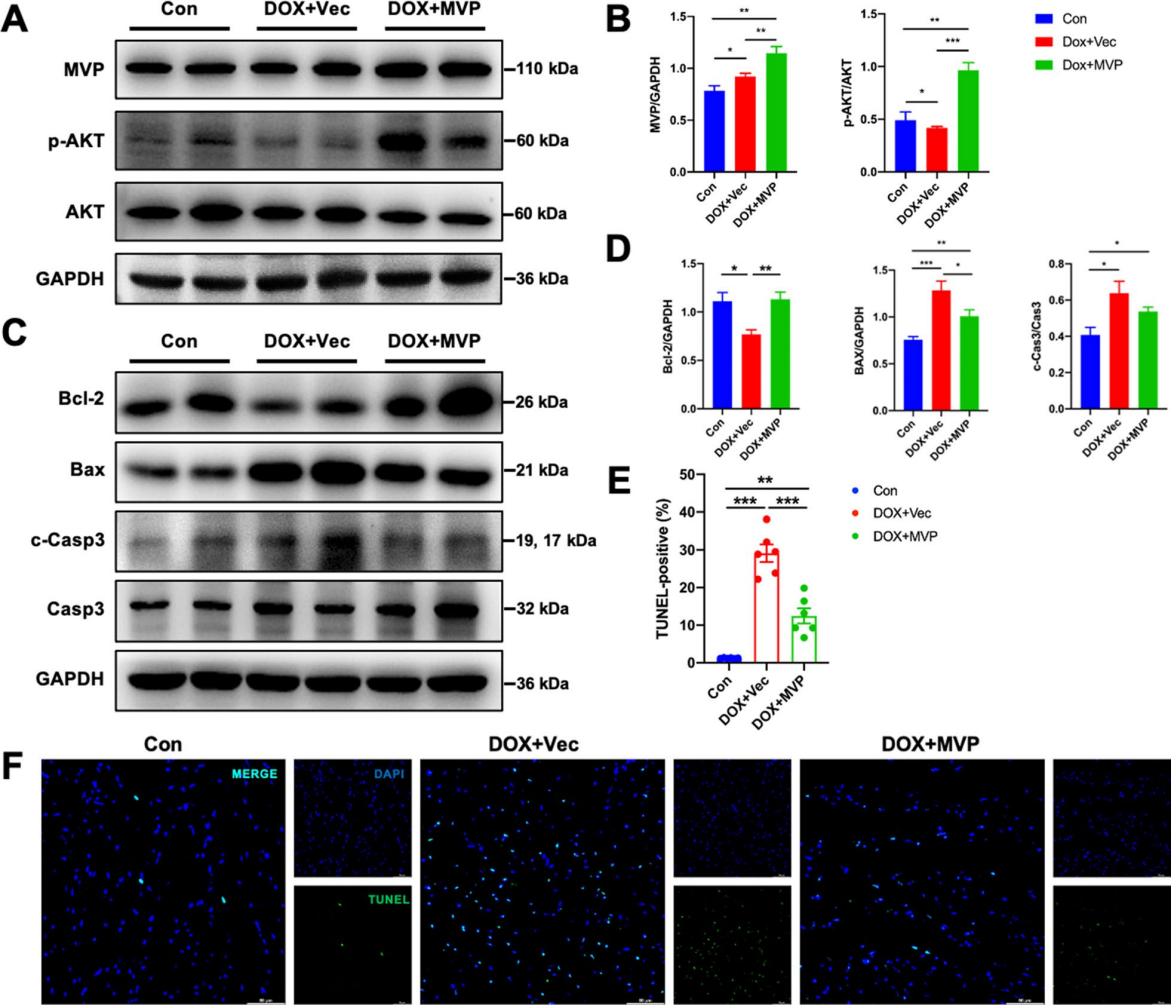
MVP suppressed cardiomyocyte apoptosis via activating AKT signaling pathway in DiCM mice. **A**, **B** Western blots and statistical results of MVP, p-AKT, and AKT in heart tissue 5 weeks after AAV9 injection. (n = 6 or 7). **C**, **D** Western blots and statistical results of Bcl-2, Bax, c-Caspase 3 and Caspase 3 in heart tissue 5 weeks after AAV9 injection. (n = 6 or 7). **E** The bar graph shows the TUNEL-positive cell ratio (%) as a cellular apoptotic index. The TUNEL-stained cells (green) were counted and normalized by the DAPI-stained cell (blue). **F** Representative fluorescence microscopic TUNEL images. (n = 6). Values represent the mean ± SEM. *P < 0.05, **P < 0.01, ***P < 0.001

Both DOX itself and excessive ROS production could induce cardiomyocyte apoptosis and contribute to the progression of cardiac dysfunction, we then assessed the role of MVP overexpression in cardiomyocyte apoptosis. As shown in Fig. 5, western blot and analysis confirmed that MVP overexpression increased the expression of Bcl-2 level, but decreased the expression of BAX and cleaved-Caspase3 level (Fig. 5C, D). AKT is an important signal transduction pathway responsible for cell survival and apoptosis [21]. Here, we observed that myocardial overexpression of MVP in vivo mitigated DOX-induced inactivation of AKT pathway (Fig. 5A, B). Collectively, these data showed that AKT signaling was responsible for MVP mediated protective role on cardiomyocyte apoptosis.

## Discussion

In this study, we identified the upregulation of MVP in DOX-induced heart failure model. Interestingly, MVP overexpression could improvement of cardiac function, inhibition of oxidative stress, fibrosis and cardiomyocyte apoptosis. MVP afford cardiac protection via the activation of AKT signaling pathway. These results highlighted that MVP may as a potential therapeutic drug target for the established DiCM and has the potential clinical translational value.

MVP is the main component of a vault, highly conserved, and the biggest ribonucleoprotein particle of the cell with a half-life of 3 days. MVP is overexpressed in multidrug-resistant cancer cells, however, whose functions are poorly understood [22–24]. These organelles are believed to be involved in several processes, including cell death resistance in cancer cells [25]. For example, Yu et al. found that MVP deficiency inhibited hepatocellular carcinoma (HCC) development with HBV or HCV infection, while forced MVP expression was sufficient to induce HCC in mice. Mechanistic studies demonstrate that MVP promotes the loss of p53 through the sequestration of interferon regulatory factor 2 (IRF2) that promotes HCC survival [14]. On the other hand, MVP regulating the proliferation signaling pathway protects cell growth from endoplasmic reticulum stress. MVP was identified as a phosphatase and tensin homolog (PTEN) -binding protein in a yeast two-hybrid screen, which shown to critical for nuclear transport of PTEN in human cells [26, 27]. PTEN is a dual-specificity phosphatase (DUSP) with a canonical tumor suppressor role in converting the lipid second messenger phosphatidylinositol- trisphosphate (PIP_3_) to phosphatidylinositol-bisphosphate (PIP_2_) that downregulates AKT signaling by reducing the output of PI3kinase at the cell membrane [28]. Our previous study also found that MVP deficiency impaired glucose tolerance and insulin tolerance in conjunction with suppressed phosphorylation of AKT [15]. To address the contribution of the MVP to AKT in the doxorubicin-induced cardiomyopathy model, we made use of AAV9 to overexpress the MVP and monitored activation of AKT in hearts. MVP overexpression alleviated cardiac dysfunction through activating AKT and decreased apoptosis.

Dilated cardiomyopathy (DCM) is characterized by left ventricular dilation which is associated with systolic dysfunction [29]. DCM is best understood as the final common response of the myocardium to diverse genetic and environmental insults [30]. Many secondary causes have been also associated with myocardial damage and the development of DCM, such as anthracyclines. Doxorubicin is a very effectively anticancer drug that is prescribed worldwide. While many patients treated with it, irrespective of age, develop insidious DCM and heart failure. The mechanism of this disease is multifactorial seeming to be associated with the generation of reactive oxygen species (ROS) and disruption of mitochondria [31, 32]. Despite progress over the past 20 years, outcomes need to be improved. Thus, this research aims to find an alternative molecular basis to treat DiCM and the identification of novel therapeutic targets, MVP.

Measurement of left ventricular (LV) size and ejection fraction (EF) remain central to diagnosis, risk stratification, and treatment, but other aspects of cardiac remodeling, especially myocardial fibrosis, inform prognosis and carry therapeutic implications [30]. Clinical research has been reported that fibrosis predicts both risk of sudden cardiac death (SCD) and the likelihood of LV functional recovery, and has significant potential to guide selection for prophylactic implantable cardioverter-defibrillator (ICD) implantation [33]. In our study, the expression of MVP was upregulated in DOX-induced cardiomyopathy which confused us at first. Then we realized that the expression of MVP may be similar to brain natriuretic peptides in heart failure which play a compensatory role. In other words, although the expression of MVP was upregulated in DiCM, which is still insufficient at the stage. So exogenous supplement of MVP may play a beneficial effect that was proved in our research. We proved that overexpression of MVP ameliorates the cardiac function in the DiCM mice model, indicating that MVP may have potential therapeutic value.

DOX mitochondrial cardiotoxicity is related to the induction of apoptosis, which has been reported in multiple studies [4]. Supporting the proapoptotic role of DOX, accumulation of proapoptotic proteins, such as BAX, in the outer mitochondrial membrane as well as a reduction in antiapoptotic proteins such as Bcl-2, and caspase-3 activation also occurs in cardiac cells after DOX treatment [19, 34]. In this study, we have shown that MVP overexpression decreased the BAX and cleaved-Caspase3 expression level, and increase Bcl-2 expression level prevented DOX-induced apoptosis of cardiomyocytes, and accompanied with increasing phosphorylation of Akt (Ser473). However, we have not blocked the Akt pathway with the Akt-specific inhibitor to assess the effectiveness, which will be an exam in the future.

## Conclusions

In the present study, we first found that DOX insult increased the MVP expression level in the heart. While the pathology upregulation of MVP may insufficient to compensate for DOX induced cardiac injury. Thus, we aimed to overexpression of MVP specifically to the myocardium to treatment established DiCM. Interestingly, MVP overexpression ameliorated cardiac function in DiCM mice model. Mechanistically, MVP-mediated AKT signaling activation in protecting cardiomyocytes from DOX-induced oxidative stress and apoptosis played a crucial role. Therefore, we highlight the importance of MVP in DiCM as a potential cardioprotective target.

## Limitations

Although paying attention to the therapeutic implication of an established DOX-induced Heart Failure mice model with MVP treatment, some limitations still existed in this study. Existing evidence indicated that MVP overexpression activated AKT signaling pathway and reduced oxidative stress and apoptosis, it is not clear how MVP activates AKT signaling. These needs to be studied in future.

## Supplementary Information

**Additional file 1:** The online version contains supplementary material including full-length blots images of western blot results.

## Data Availability

All data supporting the findings of this study are available within the published article.

## Abbreviations

AAV: Adeno-associated virus
CK-MB: Creatine kinase isoenzymes
DOX: Doxorubicin
DiCM: DOX-induced cardiomyopathy
EF: Ejection fraction
FS: Fractional shortening
LDH: Lactate dehydrogenase
MDA: Malondialdehyde
MVP: Major vault protein
ROS: Reactive oxygen species
SOD: Superoxide dismutase
TEM: Transmission electron microscopy
TUNEL: Terminal deoxynucleotidyl transferase-mediated dUTP nick end labeling

## Declarations

### ARRIVE statement

The study was carried out in compliance with the ARRIVE guidelines.
