# Supplementary figures and images for: Major vault protein attenuates cardiomyocyte injury in doxorubicin-induced cardiomyopathy through activating AKT
Source: BMC Cardiovasc Disord. 2022 Mar 4;22:77. doi: 10.1186/s12872-022-02517-9 (PMC8896232; doi:10.1186/s12872-022-02517-9)

**Supplemental Materials**

**Uncropped gels**


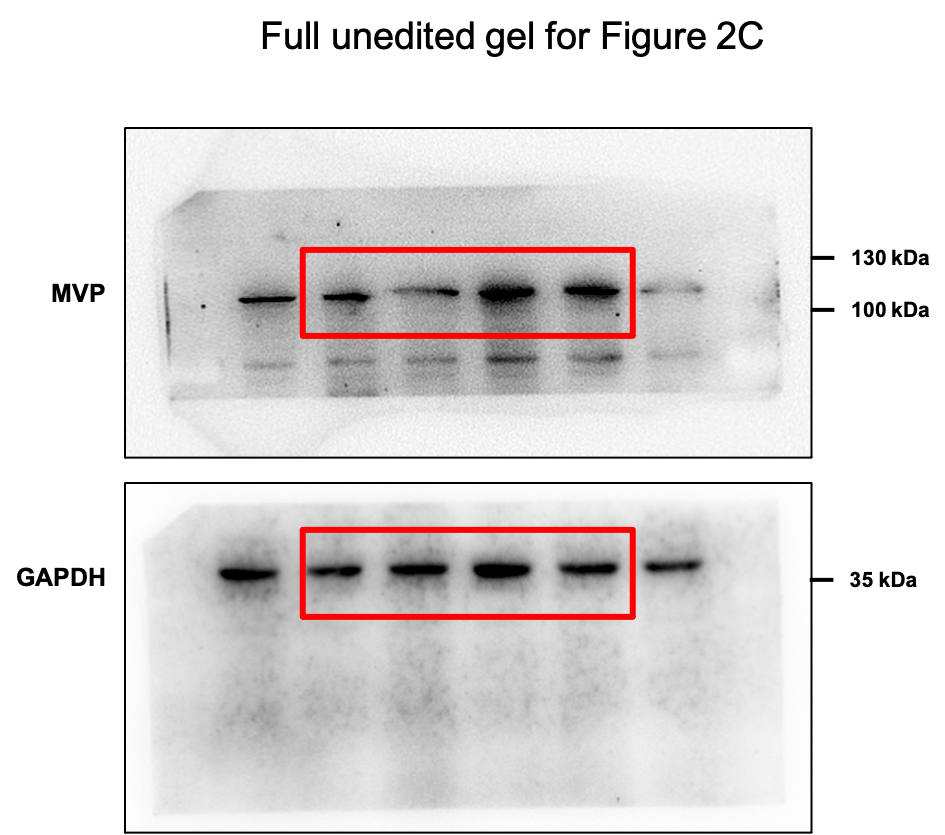


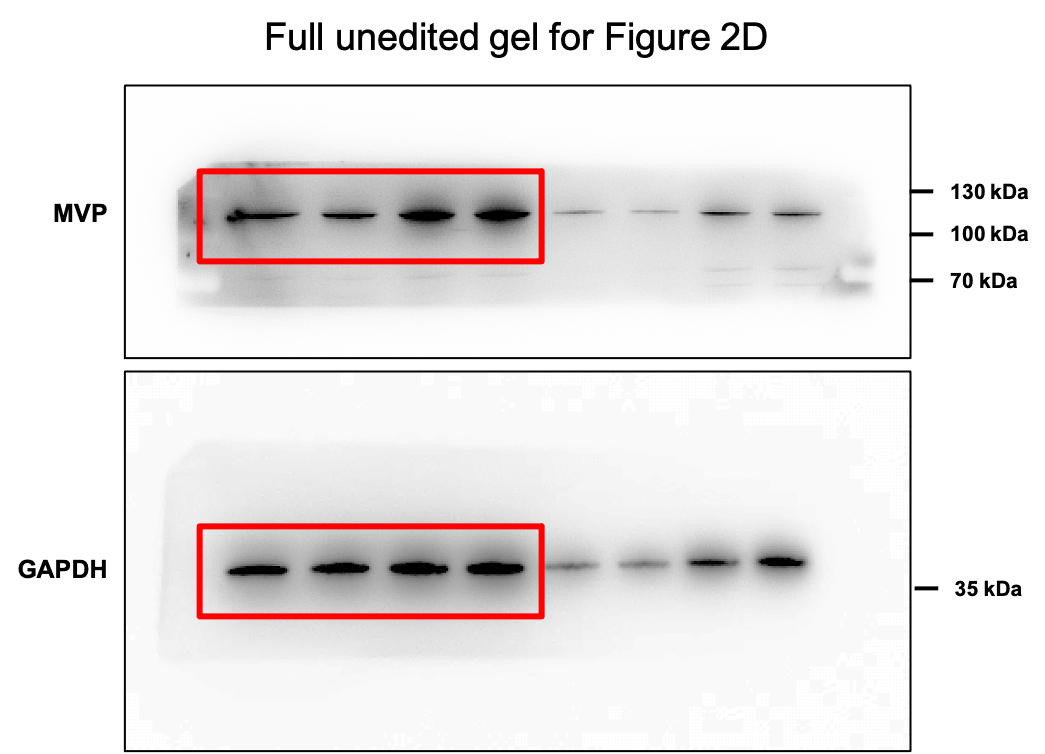


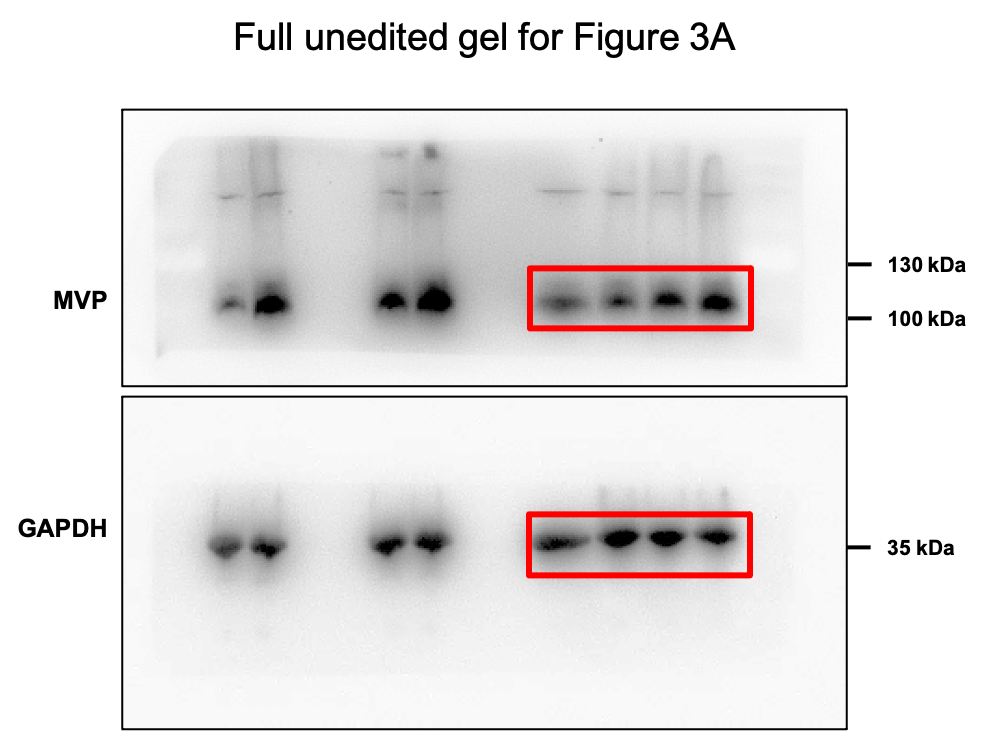


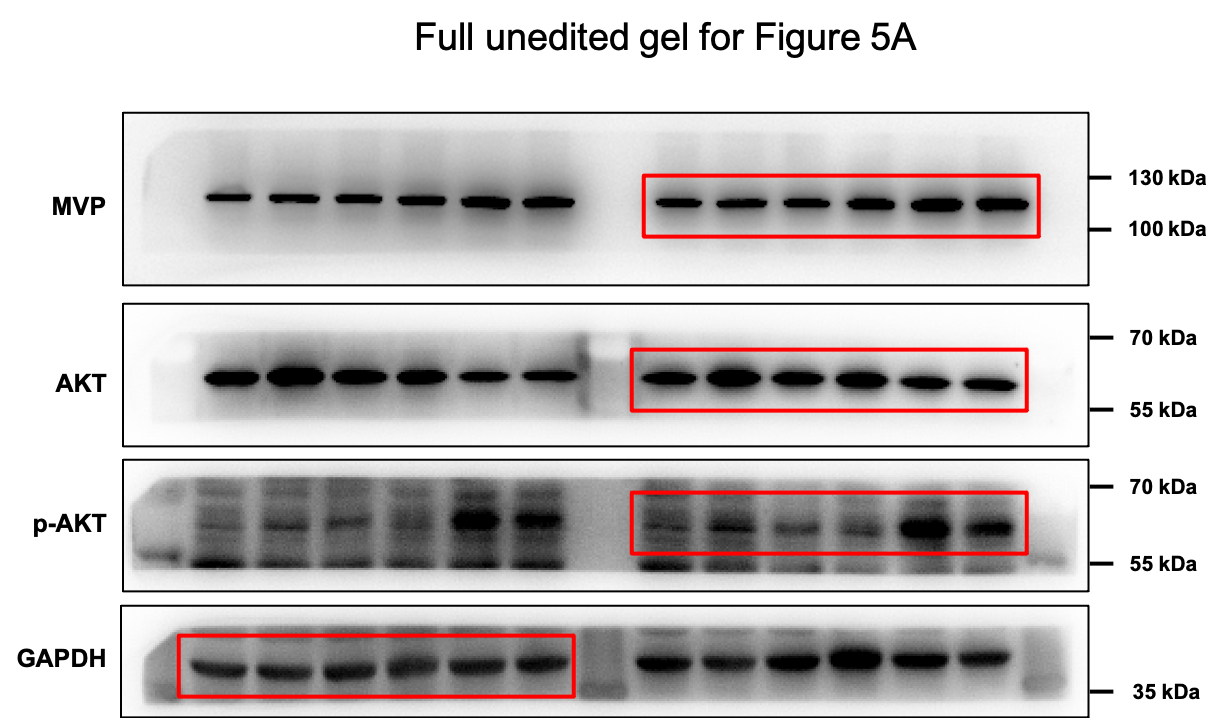


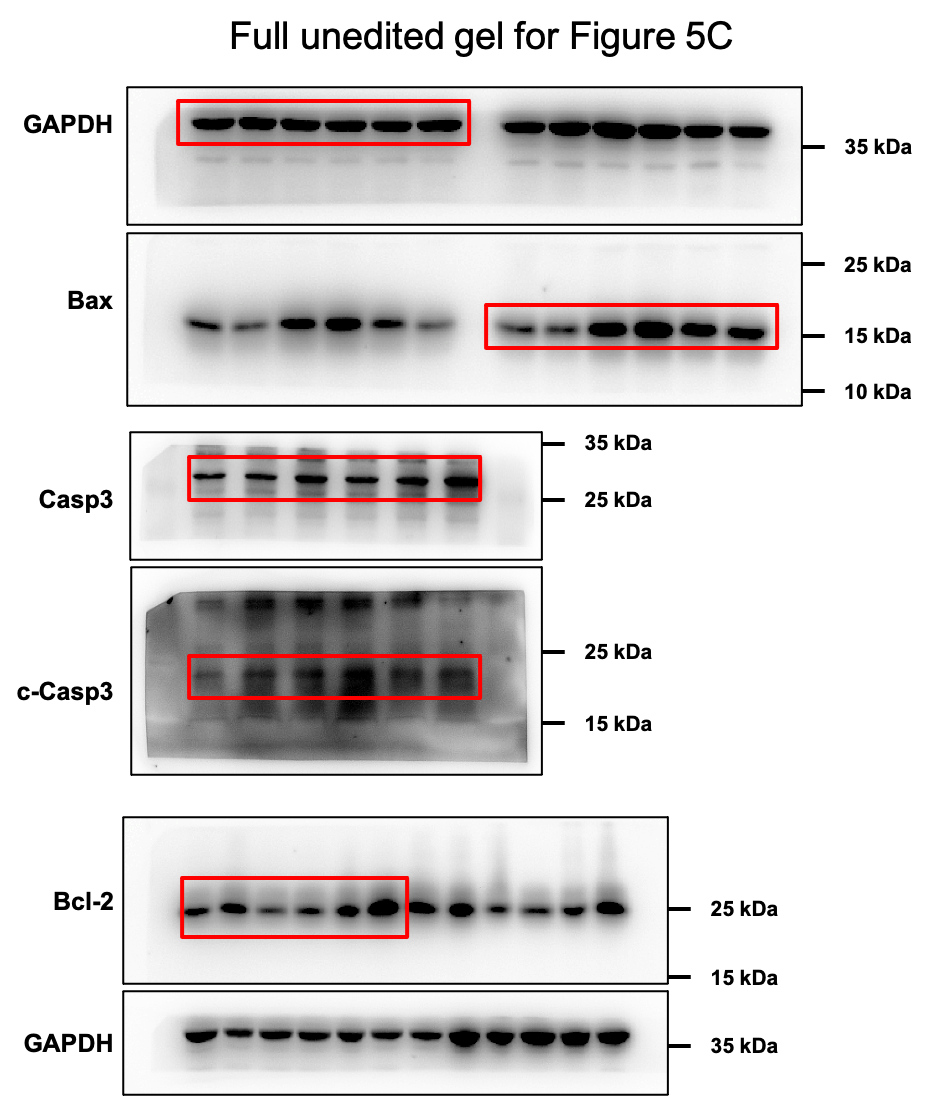

Supplement: Supplementary file 1 — Additional file 1: The online version contains supplementary material including full-length blots images of western blot results. [file 12872_2022_2517_MOESM1_ESM.docx]
